# Supplementary material for: Content-rich biological network constructed by mining PubMed abstracts
Source: BMC Bioinformatics. 2004 Oct 8;5:147. doi: 10.1186/1471-2105-5-147 (PMC528731; doi:10.1186/1471-2105-5-147)
Supplement: Additional File 5 — The original Chilibot query results of the term "long-term potentiation (LTP)" and 22 other terms, limiting the latest references analyzed to the years 1990, 1995, 2000, and 2004. [file 1471-2105-5-147-S5.bz2 › chilibotAdditionalFile5/ltp2000/html/TRKA_TRKB.html]

 


 **TRKA** and **TRKB** 
  
Found 293 abstracts in PubMed,  **30 abstracts were retrieved and analyzed**.  


---

 Search Google  |
 PDF files only 
|  EDU domain only 

---

**Interactive relationship** (e.g. stimulation, inhibition, etc)

- The neurotrophins nerve growth factor, brain derived neurotrophic factor and neurotrophin 3 bind to the tyrosine kinase trk receptors trk A  [ **TRKA** ] , trk B  [ **TRKB** ]  and trk C, respectively, with high affinity.  Ref: 11053967 Eur Neurol, 2000
- Outside the nervous system,  **TrkA**  like IR was mainly found in different epithelia,  **TrkB**  like IR in the endocrine and digestive system, and TrkC like IR in the cardiovascular and immune systems.  Ref: 10805070 Cell Tissue Res, 2000
- In addition, we observed that prolonged exposure to nerve growth factor of  **TrkA**  transfected cells did not lead to the down regulation seen with BDNF and  **TrkB** .  Ref: 10722747 J Biol Chem, 2000
- Amplification products for the full length Trk A  [ **TRKA** ]  and Trk C high affinity receptor were observed, as well as truncated isoforms for Trk B  [ **TRKB** ]  and Trk C.  Ref: 11053283 Invest Ophthalmol Vis Sci, 2000
- The resulting Trk variant recruited binding of NGF equivalent to  **TrkA** , maintained neurotrophin 3 binding equivalent to TrkC, and also bound brain derived neurotrophin, although with lower affinity compared with  **TrkB** .  Ref: 10713102 J Biol Chem, 2000
- **TrkB**  stimulated signaling proteins and induced survival and growth in a manner similar to  **TrkA** .  Ref: 10985347 Neuron, 2000

**Parallel relationship** (e.g. studied together, co-existance, homology, etc.)

- In contrast to previously studied normal retinal precursor cells, Y 79 cells not only express nerve growth factor, brain derived neurotrophic factor, neurotrophin 3, and p75, but also the corresponding high affinity receptors  **TrkA** ,  **TrkB** , and TrkC.  Ref: 10845619 Invest Ophthalmol Vis Sci, 2000
- These experiments led to the conclusion that the motives responsible for down regulation are contained in the cytoplasmic domain of  **TrkB** , and a short sequence in the juxtamembrane domain of  **TrkB**  was identified that confers nerve growth factor induced down regulation when inserted into  **TrkA** .  Ref: 10722747 J Biol Chem, 2000
- Localization of  **TRKA** ,  **TRKB** , and TRKC in a rectangular region of the rib together with surrounding soft tissues was investigated by immunostaining.  Ref: 10831935 Bone, 2000
- Neurotrophins are involved in the development and maintenance of some neuronal populations, acting through tyrosine protein kinase Trk receptors,  **TrkA** ,  **TrkB**  and TrkC.  Ref: 10839637 Anat Embryol (Berl), 2000
- To study Trk signal transduction pathways and their biological effects in NB, we stably expressed  **TrkA**  and  **TrkB**  cDNA in the human NB cell line SH SY5Y.  Ref: 10994551 Klin Padiatr< MedlineTA>Klin Padiatr, 2000
- **TrkA**  like immunoreactivity was the only one detected in all examined species, while  **TrkB**  like immunoreactivity was completely absent and TrkC like immunoreactivity was detected only in goldfish.  Ref: 10839637 Anat Embryol (Berl), 2000
- There was a decrease in trk A  [ **TRKA** ]  and C immunoreactivity in the PC of AD cases, while trk B  [ **TRKB** ]  density appeared to be unchanged.  Ref: 11053967 Eur Neurol, 2000
- At least four of the five known neurotrophins, including nerve growth factor NGF, brain derived neurotrophic factor BDNF, neurotrophin 3 NT 3, and neurotrophin 4 NT 4, and their receptors p75 NTR,  **trkA** ,  **trkB**  and trkC are present in the developing ovary.  Ref: 10963876 Mol Cell Endocrinol, 2000
- Results showed that  **trkA** , full length  **trkB** , and trkC receptors were not present in the lesion site but had a normal expression pattern in uninjured parts of the spinal cord.  Ref: 10993692 Exp Neurol, 2000
- Furthermore, our data directly reveal distinct patterns of trk receptor expression by individual sensory neurons from the time of their inception with all early arising cells initially being trkC, some subsets of whom also coexpress either  **trkA**  or  **trkB**  or both.  Ref: 11071767 Dev Biol, 2000
- The longer fragment corresponded to the classical  **trkA**  L3 transcripts while the two shorter fragments lacked sequences encoding for leucine rich motifs of the extracellular domain of  **TrkA** , similarly to the  **trkB**  L1 and L0 variants.  Ref: 10808049 J Neuroimmunol, 2000
- In contrast to  **TrkA**  signaling, p75 had a negative effect on  **TrkB**  tyrosine autophosphorylation in response to its cognate neurotrophins, brain derived neurotrophic factor and neurotrophin 4 5.  Ref: 10825163 J Biol Chem, 2000
- We examined the expression of nerve growth factor NGF and the low affinity p75 and high affinity  **trkA** ,  **trkB** , and trkC neurotrophin receptors in the trigeminal system of adult rats.  Ref: 10800206 J Neurocytol, 1999
- As a result, we observed two types of neurotrophin receptors in the bone forming area immunostaining by anti  **TRKA**  was observed in almost all bone forming cells, and staining with anti TRKC was observed in osteoblast like cells and hypertrophic chondrocytes, but no staining was observed with anti  **TRKB** .  Ref: 10831935 Bone, 2000
- Our data show that  **trkA**  and  **trkB**  are transcribed in most B cell lines of normal and malignant origin.  Ref: 10903808 Eur Cytokine Netw, 2000
- Here, 35 S riboprobes were used to determine whether pulpal afferents of adult 2 month old rats express the nerve growth factor NGF receptors, p75 NTR and  **trkA** , which are characteristic of peptidergic nociceptors, and additionally, whether these cells express receptors  **trkB**  and trkC for other members of the neurotrophin family.  Ref: 10739860 Arch Oral Biol, 2000
- Our data suggest that  **TrkA**  and  **TrkB** , once activated, provide a partial rescue from apoptosis.  Ref: 10903808 Eur Cytokine Netw, 2000
- In order to define a cellular model suitable for studying, in vitro, the molecular properties and functions of neurotrophin receptors in human lymphocytes,  **TrkA** ,  **TrkB** , TrkC and p75 expression was investigated in a panel of EBV immortalized lymphoblastoid and Burkitt lymphoma derived cell lines compared to primary B lymphocytes by RT PCR and flow cytometric analysis.  Ref: 10903808 Eur Cytokine Netw, 2000
- Four L93P, G516R, R648 C, and D668Y are missense mutations that result in amino acid substitutions at positions conserved in the TRK family, including  **TRKA** ,  **TRKB** , and TRKC.  Ref: 10982191 Hum Genet, 2000
- Introduction of  **TrkA**  and  **TrkB**  restored responsiveness of SH SY5Y cells to the ligands NGF and BDNF, respectively, and resulted in morphological differentiation.  Ref: 10994551 Klin Padiatr< MedlineTA>Klin Padiatr, 2000
- **TrkA**  and  **TrkB**  chimeric molecules were therefore expressed in HN10 cells and tested for ligand induced regulation.  Ref: 10722747 J Biol Chem, 2000
- Conditioned medium CM of parental SY5Y and SY5Y  **TrkB**  cells induced endothelial cell proliferation, but this effect was completely absent in SY5Y  **TrkA**  cells.  Ref: 11107119 Med Pediatr Oncol, 2000
- Label for  **TrkB** ... was relatively increased at and behind the optic nerve head with IOP elevation.  Ref: 10711692 Invest Ophthalmol Vis Sci, 2000
- Differential somato dendritic localization of  **TrkA** ,  **TrkB** , TrkC and p75 mRNAs in vivo.  Ref: 11043561 Neuroreport, 2000
- Alterations in trk A  [ **TRKA** ] , trk B  [ **TRKB** ]  and trk C receptor immunoreactivities in parietal cortex and cerebellum in Alzheimer s disease.  Ref: 11053967 Eur Neurol, 2000
- Evidence for an involvement of the proteasome and differential regulation of  **TrkA**  and  **TrkB** .  Ref: 10722747 J Biol Chem, 2000
- Prognostic and biological role of neurotrophin receptor  **TrkA**  and  **TrkB**  in neuroblastoma.  Ref: 10994551 Klin Padiatr< MedlineTA>Klin Padiatr, 2000
- PROCEDURE To investigate the biological effects of  **TrkA**  and  **TrkB**  expression on angiogenesis in NB, we examined the expression of angiogenic factors in the human NB cell line SY5Y and its  **TrkA**  and  **TrkB**  transfectants.  Ref: 11107119 Med Pediatr Oncol, 2000
- BACKGROUND Neurotrophins and their cognate receptors  **TrkA** ,  **TrkB**  and TrkC regulate proliferation, differentiation and death of neuronal progenitor cells and may be implicated in the progression of medulloblastoma and other primitive neuroectodermal brain tumors PNET.  Ref: 10994550 Klin PadiatrKlin Padiatr, 2000
- Among the labelled cells, 79% had positive hybridization signals for p75 NTR, 72% for  **trkA** , 34% for  **trkB** , 1% for trkC, and 77% for BDNF.  Ref: 10739860 Arch Oral Biol, 2000
- mRNA for  **TrkA** ,  **TrkB**  and p75 receptors was expressed in the injured nerve, suggesting that these specific receptors can mediate the effects of the T cell derived NTs.  Ref: 11040074 J Autoimmun, 2000
- Using antibodies directed against p75,  **trkA** ,  **trkB**  and trkC, immunolabeled neurons were present in the latero dorsal and pedunculo pontine tegmental nuclei, the peribrachial nuclei, medial and lateral pontine reticular formation, the raphe nuclei, and the locus coeruleus.  Ref: 10825475 Brain Res, 2000
- Further insight into the differences of  **TrkA**  and  **TrkB**  signaling may suggest new options for the treatment of NB.  Ref: 10994551 Klin Padiatr< MedlineTA>Klin Padiatr, 2000
- Using immunohistochemical and in situ hybridization techniques, p75,  **trkA** ,  **trkB** , and trkC receptor expression was examined throughout the spinal cord.  Ref: 10993692 Exp Neurol, 2000
- These changes were accompanied by a downregulation of  **TrkB**  and TrkC, but not  **TrkA**  or p75.  Ref: 10845619 Invest Ophthalmol Vis Sci, 2000
- METHODS Immunocytochemistry and Western blot analyses were performed using primary antibodies raised against NTs nerve growth factor NGF, brain derived neurotrophic factor BDNF, NT 3, and NT 4 5 and NT receptors  **TrkA** ,  **TrkB** , TrkC, and p75NTR.  Ref: 11053307 Invest Ophthalmol Vis Sci, 2000
- This is coupled to the ectopic expression of trk B  [ **TRKB** ]  and trk C, as well as to the continued expression of trk A  [ **TRKA** ] , and the loss of expression of p75NTR receptors.  Ref: 11027413 Prostate, 2000
- Here we describe the subcellular distribution of  **TrkA** ,  **TrkB** , TrkC and p75 mRNAs in the adult rat basal forebrain.  Ref: 11043561 Neuroreport, 2000
